# High prevalence of cesarean section births in private sector health facilities- analysis of district level household survey-4 (DLHS-4) of India

**DOI:** 10.1186/s12889-018-5533-3

**Published:** 2018-05-10

**Authors:** Priyanka Singh, Gulfam Hashmi, Prafulla Kumar Swain

**Affiliations:** 1 Nalanda Medical College, Patna, India; 2 Public Health Specialist, Lucknow, India; 3 Department of Statistics, Utkal University, Bhubaneswar, India

**Keywords:** Cesarean section, Normal delivery, Public sector, Private sector, Prevalence

## Abstract

**Background:**

Worldwide rising cesarean section (CS) births is an issue of concern. In India, with increase in institutional deliveries there has also been an increase in cesarean section births. Aim of the study is to quantify the prevalence of cesarean section births in public and private health facility, and also to determine the factors associated with cesarean section births.

**Methods:**

We analyzed data from district level household survey data 4 (DLHS-4) combined individual level dataset for 19 states/UTs of India comprising 24,398 deliveries resulting in 22,111 live births for year 2011. The percentages and Chi-square has been computed for the select variables viz. Socio demographic, maternal, antenatal care and delivery related based on type of births (CS Vs normal births). The multiple logistic regression model has been used to identify the potential risk factors associated with CS births.

**Results:**

Of 22,111 live birth analyzed 49.2% were delivered at public sector, 31.9% at private sector and 18.9% were home deliveries. Prevalence of CS births were 13.7% (95% CI; 13.0- 14.3%) and 37.9% (95% CI; 36.7- 39.0%) in the public and private sectors, respectively. Higher odds of CS births were observed with- delivery at private health facility (OR 3.79; 95% C.I 3.06-4.72), urban residence (OR 1.15; 95% C.I 1.00- 1.35), first delivery after 35 years of maternal age (OR 5.5; 95% C.I 1.85- 16.4), hypertension in pregnancy (OR 1.32; 95% C.I 1.06- 1.65) and breach presentation (OR 2.37; 95% C.I. 1.63- 3.43).

**Conclusions:**

Our findings shows that CS births are nearly three times more in private as compared to public sector health facilities.The higher rates of CS births, especially in private sector, not only increase the cost of care but may pose unnecessary risks to women (when there is no indications for CS). The government of India need to take measures to strengthen existing public health facilities as well as ensure that cesarean sections are performed based upon medical indications in both public and private sector health facilities.

## Background

Cesarean section (CS) when indicated is a live saving procedure but when performed without appropriate indications can add risk to both mother and baby [1]. Globally there is an ongoing debate on what should be the optimal rates of CS deliveries [2–5]. As per recently published WHO report, “At population level, Cesarean section rates higher than 10% are not associated with reductions in maternal and new-born mortality rates” [1]. World-wide large disparity is observed in CS rates, highest rates being reported in Latin America and the Caribbean region (40.5), followed by Northern America (32.3), Oceania (31.1), Europe (25), Asia (19.2) and Africa (7.3) [6]. In India as per District level household survey 3 (DLHS) CS rate is 28.1% in private sector and 12% in public sector health facilities [7]. This survey shows share of Cesarean deliveries in institutional births have increased in India, especially in private sector health facilities. DLHS 4 also shows similar trend in various states though country wide reports are not currently available [8].

Researchers across world have come up with various reasons for rising CS rates, important ones being “patient’s preferences”, “fear of vaginal delivery” and “social norms” [9–11]. Different rates of CS births in public and private sector health facilities suggest that non-medical factors, such as financial gain, may motivate doctors to perform CS deliveries [4, 12].

There are lack of large recent population based studies from India to explore the determinants of CS births and influence of sociodemographic, maternal, pregnancy and delivery related factors on type of delivery (Normal/ Assisted versus Cesarean Section births).

The District Level Household Survey is one of the largest ever demographic and health surveys carried out in India, with a sample size of about seven lakh households covering all districts of the country [7, 8]. The data from these surveys have been useful in setting the benchmarks and examining the progress the country has made after the implementation of reproductive child health (RCH) programme. These surveys are useful for the central and state governments in evaluation, monitoring and planning strategies. In view of the completion of 6 years of National Rural Health Mission (2005-12), there is a felt need to focus on the achievements and improvements so far.

There is a paucity of information on Cesarean section rates based on analysis of India DLHS 4 individual level dataset. In present study we try to fill this gap and create evidence from DLHS-4 individual level dataset on factors associated with Cesarean section deliveries in India.

## Methods

This study used data from District Level Household Survey (DLHS-4) individual dataset for all births of year 2011 (January 1 to 31st December) [8]. The Ministry of Health and Family Welfare (MoHFW), Government of India, conducted round 4 (DLHS-4) in 26 States and Union Territories of India during 2012-2013 (other than 9 states covered under Annual Health Survey, AHS). In the past, three rounds of DLHS have been undertaken (Round- I in 1998-99, Round-II in 2002-04, and Round-III in 2007-08). A multi-stage, stratified, probability proportional to size sample with replacement design was adopted by DLHS-4. Each district was divided into rural and urban areas. For rural areas primary sampling unit (PSU) was village and the Census of India 2001 was the sampling frame. For urban areas PSU were NSSO Urban Frame Survey (UFS) blocks. UFS blocks in each district have been stratified into million-class cities and non-million class cities and allocation of sample was proportional to relative sizes. Twenty-five households have been selected from each rural and urban PSU. Further detailed description of sample methodology and survey process of DLHS-4 has been mentioned elsewhere [8] ^.^The survey obtained detailed information on socio demographic details, maternal characteristics, pregnancy and delivery from ever married woman aged 15-49 years.

As available in public domain (accessed from http://www.iipsindia.ac.in/) at the time of analysis, combined individual data set for following 19 states/ UTs (Himachal Pradesh, Punjab, Haryana, Sikkim, Arunachal Pradesh, Nagaland, Manipur, Mizoram, Tripura, Meghalaya, West Bengal, Maharashtra, Andhra Pradesh, Karnataka, Goa, Kerala, Tamil Nadu and Telangana) were used for analysis in present study.

### Definition of variables

#### Outcome variable

We defined the type of delivery (CS or normal) as outcome variable.

#### Explanatory or independent variables

A wide range of independent variables were used for this analysis. The potential select variables were: maternal age, place of residence, women’s education, caste, age at first delivery, wealth index quartiles, received four or more ANC and abdomen checked, blood pressure measured, blood test for haemoglobin, urine test done at least once during pregnancy, Ultrasound done at least once during pregnancy, main place of receiving ANC, complications during pregnancy pallor/ weakness/ giddiness, weak or no movement of foetus, abnormal position of foetus, high blood pressure, bleeding during pregnancy and delivery complications prolonged labour, obstructed labour, excessive bleeding, breach presentation and convulsion and place of delivery place of delivery has been divided into public sector and private sector health facilities as categorised in DLHS questionnaire (refer Table 1).

**Table 1:** Subcategories for place of delivery in public and private sector health facilities

Place of delivery	Sub categories
Public sector health facility	Government/municipal hospital, government dispensary, urban healthcare, urban family welfare center, community health center, rural hospital, primary health center, sub center, and village clinic by auxiliary nurse midwife.
Private sector health facility	Private hospital or clinic, and NGO hospital or clinic.
Home	Home, parent’s home, work place, on way to hospital, others

Variables of interest were taken from “Ever Married Woman’s Questionnaire”. The analysis is based on information regarding the latest birth of ever-married women aged 15-49 years, who had given live birth in year 2011 (1st January to 31st December) and reported the “type of delivery”. Cesarean section rates were calculated as number cesarean section births out of total live birth for year 2011.

### Statistical analysis

The proportion and percentages were computed and univariate Chi-square test was performed. A multiple logistic regression was used to determine the significant predictors associated with the CS births. Since the outcome variable is a dichotomous variable the multiple logistic regression model was a natural choice. The predictors which are found to be significant in univariate analysis are included in the regression model, the final model was adjusted for all the confounding variables. The results of the regression model have been presented in the form of odds ratio (OR) along with its 95% confidence intervals (CI). The analysis accounted for complex cluster sampling design; hence a complex sample analysis was carried considering the study design and sampling weight of DLHS 4. All the statistical analysis was carried out using SPSS version- 20.0. In all case the *P*-value < 0.05 was considered as significant.

## Results

### Type of delivery (refer Fig. 1)

Of total 24,398 deliveries, 90.6% (*N* = 22,111) were live births and rest 9.4% (*N* = 2287) resulted in still births. 81.1% (*N* = 17,934) of the total 22,111 live births were institutional (49.2 and 31.9% at public and private sector health facility respectively) and 18.9% (*N* = 4177) delivered at home.

**Fig. 1:**
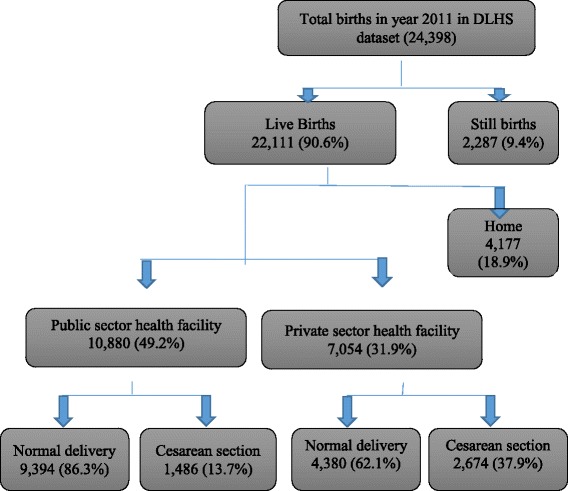
Flow chart for births analysed in this study

Prevalence of CS births were in public sector health facilities was13.7% (95% CI; 13.0- 14.3%) as compared to 37.9% (95% CI; 36.7- 39.0%) in private sectors health facilities.

### Socio demographic and maternal factors (refer Table 2)

This study shows as the maternal age advances proportion of live births by cesarean section as compared to normal delivery increased from 18.5% in woman aged < 19 years to 28.6% in age group > 35 years (*p* value< 0.001).

**Table 2:** Sociodemographic and maternal factors based on type of delivery

Variables	Categories	Normal	Caesarean Section	Chi-Square	*P*-Value
		N (%)	N (%)		
Age of Mother	< = 19 Years	330 (81.5)	75 (18.5)	80.62	0.001
	20 - 24 Years	4874(80.3)	1196 (19.7)		
	25 - 34 Years	7790 (75.1)	2576 (24.9)		
	> = 35 Years	780 (71.4)	313 (28.6)		
Residence	Rural	8572 (80.5)	2074 (19.5)	202.91	0.001
	Urban	5202 (71.4)	2086 (28.6)		
Wealth Index	First Quartile	3059 (87)	458 (13)	401.62	0.001
	Second Quartile	3575 (80.2)	880 (19.8)		
	Third Quartile	3578 (73.6)	1284 (26.4)		
	Fourth Quartile	3558 (69.8)	1538 (30.2)		
Caste	ST/SC	5857 (82.7)	1226 (17.3)	231.6	0.001
	Others	7194 (73)	2701 (27)		
Age at first delivery	< = 19 Years	4387 (84.9)	783 (15.1)	522.76	0.001
	20 - 24 Years	7053 (77.2)	2084 (22.8)		
	25 - 34 Years	2286 (64.8)	1243 (35.2)		
	> = 35 Years	44 (46.8)	50 (53.2)		
Maternal education	Any Schooling	11,496 (75)	3832 (25)	192.22	0.001
	No Schooling	2276 (87.4)	328 (12.6)		

Data missing: wealth index for 4; Age at first delivery for 8; maternal education for 2

It was also observed that the CS births increases with advancing age of the women at the time of first delivery from 15.1% in aged less than 19 years to 53.2% amongst aged greater than 35 years (*P* value < 0.001). Higher proportion of live birth deliveries in urban areas were through cesarean section (28.6%) compared to 19.5% in rural areas, (*p* value < 0.001). CS births were higher among women with any schooling (25%) as compared to no schooling (12.6%) (P value < 0.001). SC/ST caste women reported less deliveries by CS as compared to other castes (17.3% Vs 27%).

Proportion of CS births significantly increased from lower to highest wealth index quartile (*p* value< 0.001) (13, 19, 26, 4, 30.2% in first, second, third, fourth quartile respectively).

### Pregnancy and delivery related factors (refer Table 3)

It was observed that delivery by cesarean section was 26.9% in women who received 4 or more ANC and 18.0% in women who received less than 4 ANC (*p* value< 0.001).

**Table 3:** Pregnancy and delivery related factors based on type of delivery

Variables	Categories	Normal	Caesarean	Chi-Square	*P*-Value
Antenatal care related		N (%)	N (%)		
4 + ANC	Yes	8236 (73.1)	3035 (26.9)	131.01	0.001
	No	3413 (82)	749 (18)		
Received abdomen check BP Blood Test and Urine Test at least once	Yes	12,106 (75.6)	3913 (24.4)	127.63	0.001
	No	1659 (87.1)	245 (12.9)		
USG done	Yes	9099 (73)	3360 (27)	325.87	0.001
	No	4666 (85.4)	798 (14.6)		
Main place of ANC	Public	8163 (83.3)	1641 (16.7)	732.03	0.001
	Private	4319 (64.9)	2335 (35.1)		
	Home	156 (80.4)	38 (19.6)		
Complications during pregnancy					
Pallor/ weakness/ giddiness	Yes	1515 (75.4)	495 (24.6)	2.61	0.106
	No	12,258 (77)	3664 (23)		
Weak or no movement of foetus	Yes	273 (72.4)	104 (27.6)	4.17	0.041
	No	13,500 (76.9)	4055 (23.1)		
Abnormal position of fetus	Yes	179 (73.4)	65 (26.6)	1.65	0.199
	No	13,594 (76.9)	4094 (23.1)		
High Blood Pressure	Yes	527 (64.9)	285 (35.1)	67.67	0.001
	No	13,246 (77.4)	3874 (22.6)		
Bleeding	Yes	143 (73.3)	52 (26.7)	1.34	0.248
	No	13,630 (76.8)	4107 (23.2)		
Delivery complications					
Convulsion	Yes	370 (61.7)	230 (38.3)	79.77	0.001
	No	13,400 (77.3)	3930 (22.7)		
Excessive bleeding	Yes	718 (76.5)	221 (23.5)	0.06	0.803
	No	13,052 (76.8)	3939 (23.2)		
Prolonged labour	Yes	1085 (73.3)	396 (26.7)	11.34	0.001
	No	12,685 (77.1)	3764 (22.9)		
Breach presentation	Yes	243 (55.9)	192 (44.1)	109.67	0.001
	No	13,527 (77.3)	3968 (22.7)		
Obstructed labour	Yes	1350 (75.5)	439 (24.5)	2.01	0.158
	No	12,420 (76.9)	3721 (23.1)		
Place of delivery	Private	4380(62.1)	2674(37.9)	1413	0.001
	Public	9394(86.3)	1486(13.7)		

Data missing: four or more antenatal care visits for 2501; Received abdomen check BP Blood Test and Urine Test at least once for 11; USG at least once for 11; Main place for 1282; Pallor/ weakness/ giddiness for 2; Weak or no movement of foetus for 2; Abnormal position of fetus for 2; High Blood Pressure for 2; Bleeding for 2; convulsion for 4; Prolonged labour for 4; Breach presentation for 4; Obstructed labour for 4

Similar pattern was seen with those who received abdomen checkup, blood pressure checked, blood test for hemoglobin and urine test done at least once during pregnancy. Overall, it was observed that the CS births were 24.4% in women who received abdomen checkup, blood pressure checked, blood test for hemoglobin and urine tested at least once during pregnancy as compared 12.9% in women who did not receive these checkup even once during pregnancy (*p* value< 0.001).

Women who reported ultra sound at least once had 27% CS births compared to 14.6% in women who did not report ultra sound test (*p* value< 0.001).

35.1% women receiving ANC primarily at private hospitals delivered by cesarean section compared to 16.7% women who received ANC primarily at public hospitals and 19.6% in women who received ANC at home.

Woman with high blood pressure during pregnancy had significantly higher proportion of C S births than those who had normal blood pressure (35.1% Vs 22.6%).Woman with delivery related complications of convulsion, prolonged labour and breach presentation had significantly higher proportions of CS births (*p* value< 0.001).

### Multiple logistic regression (refer Table 4)

Table 4 shows the results of the predictor associated with caesarean section births. Particularly the socio-demographic and maternal related variables viz., residence, wealth index quartile and mothers age at first delivery are found to be statistically significant (*P*-value< 0.05). The odds of CS births were higher for women who belonged to urban areas (OR 1.15, 95% CI; 1.00-1.35). The Mothers’ first pregnancy at aged more than 35 years had 5.51 times more likely to have CS delivery than those were younger (age < 19 years) at their first pregnancy (OR 5.51; 95% CI 1.85-16.40). The women who belong to richer wealth index quartile is having more risk of CS delivery as compared to poor wealth index quartile (OR 1.19, 95% CI; 1.01-1.57).

**Table 4:** Association of socio-demographic, maternal, pregnancy and delivery related variables with Cesarean section deliveries using multiple logistic regression

Variables	Categories	Total	Delivered by C section	
		N = 17,934 (% of total)	*n* = 4160 (% of N)	Odds ratio (95% CI)
Socio-demographic and maternal				
Age of Mother	< = 19 Years	405 (2.26)	75 (1.80)	1.38 (0.76-2.50)
	20 - 24 Years	6070 (33.85)	1196 (28.75)	1.19 (0.97-1.45)
	> = 35 Years	1093 (6.09)	313 (7.52)	1.2 (0.81-1.76)
	25 - 34 Years	10,366 (57.8)	2576 (61.92)	1
Residence	Urban	7288 (40.64)	2086 (50.14)	1.15 (1.00-1.35)
	Rural	10,646 (59.36)	2074 (49.86)	1
Wealth index	Fourth	3517 (19.62)	1538 (36.97)	1.19 (1.01-1.57)
	Third	4455 (24.85)	1284 (30.87)	1.08 (0.83-1.42)
	Second	4862 (27.12)	880 (21.15)	1.32 (1.01-1.73)
	First quartile	5096 (28.42)	458 (11.01)	1
Caste	Others	9895 (55.17)	2701 (64.93)	1.07 (0.90-1.26)
	ST/SC	7083 (39.49)	1226 (29.47)	1
Maternal education	Any Schooling	15,328 (85.47)	3832 (92.12)	1.01 (0.74-1.36)
	No Schooling	2604 (14.52)	328 (7.88)	1
Age at first delivery	> = 35 Years	94 (0.52)	50 (1.20)	5.51 (1.85-16.40)
	25 - 34 Years	3529 (19.68)	1243 (29.88)	2.49 (1.87-3.32)
	20 - 24 Years	9137 (50.95)	2084 (50.10)	1.43 (1.15-1.77)
	< = 19 Years	5170 (28.83)	783 (18.82)	1
Antenatal care related				
4 + ANC	Yes	11,271 (62.85)	3035 (72.96)	1.23 (1.00-1.49)
	No	4162 (23.21)	749 (18)	1
Received abdomen check + BP check+ Blood Test + Urine Test at least once	No	1904 (10.62)	245 (5.89)	1.28 (0.68-2.39)
	Yes	16,019 (89.32)	3913 (94.06)	1
USG done	Yes	12,459 (69.47)	3360 (80.77)	1.37 (1.09-1.74)
	No	5464 (30.47)	798 (19.18)	1
Complications during pregnancy				
Weak or no movement of foetus	Yes	377 (2.1)	104 (2.5)	1.01 (0.74-1.36)
	No	17,555 (97.89)	4055 (97.48)	1
Abnormal position of fetus	Yes	244 (1.36)	65 (1.56)	1.11 (0.75-1.66)
	No	17,688 (98.63)	4094 (98.41)	1
High BP	Yes	812 (4.53)	285 (6.85)	1.32 (1.06-1.65)
	No	17,120 (95.46)	3874 (93.13)	1
Bleeding	Yes	195 (1.09)	52 (26.67)	1.24 (0.83-1.87)
	No	17,737 (98.9)	4107 (23.15)	1
Delivery complications				
Convulsion	Yes	600 (3.35)	230 (5.53)	1.15 (0.84-1.58)
	No	17,330 (96.63)	3930 (94.47)	1
Excessive bleeding	Yes	16,991 (94.74)	3939 (94.69)	1.55 (1.17-2.04)
	No	939 (5.24)	221 (5.31)	1
Prolonged labour	Yes	16,449 (91.72)	3764 (90.48)	1.05 (0.83-1.35)
	No	1481 (8.26)	396 (9.52)	1
Obstructed labour	Yes	16,141 (90)	3721 (89.45)	1.41 (1.11-1.78)
	No	1789 (9.98)	439 (10.55)	1
Breach presentation	Yes	435 (2.43)	192 (4.62)	2.37(1.63-3.43)
	No	17,495 (97.55)	3968 (95.38)	
Place of delivery				
Place of Delivery	Private	7054 (39.33)	2674 (64.28)	3.79 (3.06-4.72)
	Public	10,880 (60.67)	1486 (35.72)	1

The predictors like more than 4 ANC check-up, ultrasound diagnosis, high BP, Excessive bleeding, breach presentation and obstructed labour were significantly associated with CS deliveries (*P*-value< 0.05). There is an increase in CS births with increase in the number of ANC visits. The women who had more than 4 ANC visit had 1.23 times more likely of CS delivery as compared to those who did not make any ANC visit. Complaint of high BP during pregnancy had significantly higher odds of CS births (OR 1.32; 95% CI 1.06-1.65). The history of delivery complication especially, breach presentation (OR 2.37; 95% CI 1.63-3.43), obstructed labour (OR 1.41 95% CI 1.11-1.78) and excessive bleeding during delivery (OR 1.55; 95% CI 1.17-2.04) had higher odds of CS Births. Also, delivery at a private sector health facility had 3.79 times higher odds of CS births (OR 3.79, 95% CI 3.06-4.72) as compared to delivery at public sector health facility.

## Discussion

**Fig. 2:**
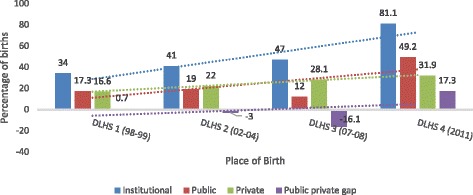
Trends in Institutional deliveries in India evidence from District level Household Surveys (DLHS)

Promoting Institutional deliveries is an important intervention of Government of India to decrease maternal morbidity and mortality. Findings show that although higher proportion of woman deliver at public sector, CS births are more in private sector health facilities. India has observed a sharp rise in institutional deliveries (Fig. 2), from 47% (DLHS 3, 2007-08) to 81.1% in current study (DLHS 4, 2011-12). This sharp increase after launch of the Janani Suraksha Yojana (JSY) and Janani Shishu Suraksha Karyakram (JSSK) schemes could be attributed to large extent to these programme being able to increase institutional births and hence decrease home deliveries [13]. Interestingly, earlier higher proportion of institutional deliveries took place in private sector whereas present study shows public sector is now bigger contributor to institutional births. Result also shows of all those delivered in private sector nearly 38% delivered by CS as compared to only 14% in public sector. Various studies have shown that there are multiple factors which influence a woman’s decision to deliver by cesarean section [14]. For profit making some private sector providers may perform unwanted CS deliveries, while woman themselves can also opt for CS delivery due to fear of pain or they believe CS deliveries are safer [15–19]. In current study, CS rates in private health facilities rates are much greater than the WHO recommended maximum limit of 15% for CS births [3, 20]. Other studies have also confirmed that there is greater prevalence of CS births in private sector health facilities as compared to public sector health facilities [21–25]. Though there has been a growing demand to revisit these rates, still these rates being selectively high in private sector is area of concern. Flagship program of Government of India. “Pradhan Mantri Surakshit Matritva Abhiyan” (PMSMA) can be utilised so as to reduce service provider induced increased CS rate of private sector.

Significantly higher proportion of woman with age more than 35 years and less than 19 years were associated with increased proportion of cesarean section (*p* value< 0.001). Others studies have also shown that greater maternal age as an important factor associated with cesarean births because other medical conditions like hypertension, diabetes being more prevalent at higher age group [26, 27].

More cesarean section births took place in urban woman as compared to the ones who resided in rural areas. More accessibility to medical intervention in urban areas, presence of more health facilities and insurances in urban areas can be probable reasons [28].

Wealthier woman, belonging to higher caste group and having some schooling and more likely to deliver by cesarean section (*p* value< 0.001). Cesarean section seems to be a choice method for woman who can afford it rather than being a procedure for safe delivery when medically indicated [29, 30]. Woman with lower socioeconomic status might cannot afford or do not have access to health facilities which are equipped to perform cesarean delivery. Other studies on CS births in developing countries have also supported this finding [31].

The finding of higher cesarean rates amongst mothers whose first delivery is after age of 35 years is matter of concern, since this contributes to increased further CS deliveries, considering that history of CS delivery is a relative indication for CS in subsequent delivery [32, 33].

Findings shows greater number of antenatal care visits being associated with higher CS Birth rates. Women who had four or more antenatal check-ups were significantly more likely to have a CS births (OR 1.23 95% CI 1.0-1.5). Antenatal care is the care given to pregnant woman by health care provider for safe pregnancy and child birth. Antenatal care is the ideal time to counsel and prepare pregnant woman for normal delivery (if there are no medical indications for CS), since this is the time she can interact with her care giver and plan safe delivery [19, 34]. Other studies have also shown this finding, as greater number of ANC visits allows more interaction between the care provider and pregnant woman, which might influence her to provider induced CS delivery [35, 36]. This finding has programmatic implications, ANC visit can be an opportunity to increase institutional deliveries and counsel mother regarding indications of cesarean section and risk involved [16, 19].

Ultrasound imaging done at least once during current pregnancy was significantly associated with higher risk of CS births. Ultrasound imaging is an essential component of antenatal care services. Also recommended by WHO for safe pregnancy and foetal monitoring [35, 36]. It helps to assess foetal growth, congenital anomalies or maternal complications if any at a very early stage so that the detected condition can be managed at the earliest [37, 38].

Study shows higher risk of cesarean section in case mother had complications of high blood pressure during pregnancy. Antenatal screening for high blood pressure and timely management of such mothers can bring down unwanted CS procedures. As expected, the chance of CS is higher among those who have experienced complications during delivery (excessive bleeding, obstructed labour, breach presentation). Literature also supports this finding as these complications may lead to higher risk to both mother and baby hence cesarean section needed as live saving procedure [4, 39].

## Conclusions

The caesarean section is a globally recognised maternal health-care indicator. Unnecessary caesarean sections also pulls resources away from other services in overloaded and weak health systems. In India, CS rates are high in private sector health facilities, though their share in total deliveries is much lesser as compared to public sector health facilities. Hence there is need for strengthening of existing public health facilities especially, emergency obstetric care services so that if medically indicated live saving surgical procedure can be performed in public sector itself rather than drifting woman towards profit driven private sector health facilities. The government of India need to take measures to ensure that caesarean sections are performed based upon medical indications. Appropriate mechanism and specific opportunities for awareness generation and provider education, focused upon populations which are likely to prefer cesarean section, should be developed. Appropriate monitoring and accountability systems should be developed at both national and state levels in India.

## Key messages

- Though higher proportions of institutional deliveries take place at public sector health facilities, Cesarean Section births are nearly three times higher in private sector health facilities.
- Non-medical determinants of Cesarean Section like place of delivery, household wealth index, residence, age of mother at first delivery, number of ANC can be looked upon in order to keep check on unwanted CS births.
- Antenatal Care can be utilised as an opportune time to explain the indication and hazards associated with CS birth.

## Abbreviations

AHS: Annual Health Survey
ANC: Antenatal care
BP: Blood pressure
BP: Blood pressure
C I: Confidence interval
CS: Cesarean section
DLHS: District level household survey
JSSK: Janani Shishu Suraksha Karyakram
JSY: Janani Suraksha Yojana
OR: Odds ratio
PMSMA: Pradhan Mantri Surakshit Matritva Abhiyan
RCH: Reproductive child health
UT: Union Territories
